# Assessment of SIP Buildings for Sustainable Development in Rural China Using AHP-Grey Correlation Analysis

**DOI:** 10.3390/ijerph14111292

**Published:** 2017-10-25

**Authors:** Libiao Bai, Hailing Wang, Chunming Shi, Qiang Du, Yi Li

**Affiliations:** 1 School of Economics and Management, Chang’an University, Middle Section of South Second Ring Road, Xi’an 710064, China; LB.Bai@chd.edu.cn (L.B.); hailing711@163.com (H.W.); q.du@chd.edu.cn (Q.D.); 2 Lazaridis School of Business and Economics, Wilfrid Laurier University, Waterloo, ON N2L3C5, Canada; 3 School of Civil Engineering, Chang’an University, Middle Section of South Second Ring Road, Xi’an 710064, China; liyi0224@hotmail.com

**Keywords:** SIPs, rural residence, low-carbon building, sustainable development

## Abstract

Traditional rural residential construction has the problems of high energy consumption and severe pollution. In general, with sustainable development in the construction industry, rural residential construction should be aimed towards low energy consumption and low carbon emissions. To help achieve this objective, in this paper, we evaluated four different possible building structures using AHP-Grey Correlation Analysis, which consists of the Analytic Hierarchy Process (AHP) and the Grey Correlation Analysis. The four structures included the traditional and currently widely used brick and concrete structure, as well as structure insulated panels (SIPs). Comparing the performances of economic benefit and carbon emission, the conclusion that SIPs have the best overall performance can be obtained, providing a reference to help builders choose the most appropriate building structure in rural China.

## 1. Introduction

From the United Nations Framework Convention on Climate Change (UNFCCC) to the Copenhagen Accord, and most recently, Conference of the Parties 21 (COP21) in Paris, many countries have been seeking measures to maintain both social and economic development and environmental improvement for sustainability. Global warming has been a severe environmental problem for human beings in recent decades. It is widely known that greenhouse gases, especially carbon dioxide (CO_2_), is blamed as the main cause of global warming [1]. As the primary contributor of global greenhouse gas (GHG) emissions, the construction industry plays a significant role in global warming. The Intergovernmental Panel on Climate Change asserts that the building sector contributed 40% to the total energy consumption and 25% to the global total CO_2_ emissions [2].

In China, much more attention has been paid to the GHG emissions from cities, owing to its accelerated urbanization. However, China still has more than 600 million people, occupying 24 billion square meters of building space, in vast rural areas [3]. Based on the latest projections of the World Bank, the total population of China will be approximately 1.5 billion in 2030 [4]. Even if China can maintain a stable urbanization rate and eventually achieve a two-thirds urbanization ratio, there will still be more than 500 million people living in rural areas within the next two decades. Even with fast urbanization, rural areas will still account for 38% of the total population in 2030 and 27% in 2050 [5]. Thus, promoting and accelerating the sustainable development of rural housing has strategic meaning for improving the living conditions of rural people, for reducing energy consumption, for improving environmental quality, and for promoting economic development [6].

Green building emerged from the green movement around 1970s–1980s as a solution for meeting building demand while reducing the construction industry’s energy consumption and carbon emissions [7]. Green building is a practice of creating and using healthier and more resource-efficient models of construction, renovation, operation, maintenance and demolition [8,9], which is defined as “those embracing the principles of lower environmental impact through greater energy efficiency, lower energy demand, reduced water usage, improved indoor quality and minimizing construction waste” [10]. Studies have shown that the greening technologies and design applied in green building can increase the efficiency of buildings by up to ten times in terms of resource utilization (Green building: project planning & cost estimating 2011) [11]. Therefore, green building in rural areas can help to improve energy efficiency and living conditions, and decrease carbon emissions.

In existing studies on rural green building, some researchers have analyzed the major factors influencing carbon emission and proposed corresponding measures for reducing energy consumption and carbon emissions [9,12,13,14,15]. Some researchers focused on energy and environment of rural construction [6,16,17] and analyzed energy efficiency and indoor thermal environment. However, there is lack of a systematic investigation of rural architectural structures that influence energy efficiency and carbon emission. Though the alternatives of low-carbon building are various, the structure of rural houses is currently simple. The living space per capita of rural residents increased from 8.1 m^2^ in 1978 to 34.1 m^2^ in 2010, and rural housing quality also improved and diversified [18]. 70% of new constructed rural houses in 2010 had a reinforced concrete structure, versus 26.2% with masonry-timber structure [19]. Because of their high operating costs and carbon emissions, buildings constructed by brick and concrete are not consistent with the goal of sustainable development. It is imperative to find one or more alternative structures.

For promoting green building in rural areas, a new structure, structure insulated panels (SIPs), is argued for in this paper. SIPs, which are structural sandwich panels consisting of a foam plastic insulation core securely bonded between two structural panel facings, such as oriented strand board (OSB), are one of the fastest growing products in the U.S. housing construction industry, due in part to their energy and construction efficiencies, and their distinctive structural performance characteristics.

Facing materials used in SIPs can be oriented strand board (OSB), plywood, metal, cementitious, magnesium, plastic, or other structural panel products. Core materials used in SIP construction include EPS (expanded polystyrene), XPS (extruded polystyrene), urethanes, isocyanates, and other insulating materials. Now, the current and usual material used for SIPs in China is OSB, which is a recycled material with high utilization of wood than solid wood and the core materials is that has no damage to ozone depletion. These components are used in commercial and residential construction projects worldwide [20]. Moreover, it is very appropriate for low-rise building which is also the main type of rural residence.

Although SIP buildings have superior performance over traditional brick-concrete houses in the aspect of structure, construction and thermal insulation, economic cost must be considered in a developing country, such as China. Furthermore, SIP buildings should meet the current requirement of low-carbon development in China. So, in this paper, the economic benefit and carbon emission benefit are selected as the basic performance indices. These will be used, in combination with life-cycle assessment, net present value and initial investment cost, to make a comprehensive and systematic analysis and evaluation for SIP buildings. Our methodology will be the AHP-Grey Correlation Analysis.

This work proves that the SIP structure is more suitable for rural areas in order to reduce carbon emissions, improve energy efficiency and achieve sustainable development. The structure of the rest of this paper is organized as follows. AHP-Grey Correlation Analysis is proposed in Section 2, including economic evaluation and low-carbon evaluation. Based on this method, the Section 3 compares four building structures, namely, brick and concrete structure, timber-framed structure, SIPs, and light-steel structure, using a case study. Section 4 elaborates and discusses the results from the case study. The conclusions, suggestions and future research directions are presented in Section 5.

## 2. Methods

### 2.1. Analytic Hierarchy Process—Grey Correlation Analysis

The Analytic Hierarchy Process (AHP) is a general theory of measurement, developed by Saaty in the 1970s [21,22,23] and used for multi-criteria decision making [24,25]. Problems that include multiple objectives, principles or levels can be solved by this methodology. It works by structuring a decision problem as a hierarchy with an overall goal, a group of alternatives, and of a group of criteria that link the alternatives to the goal [26]. Grey-correlation analysis is a part of grey system theory, which is suitable for solving complicated inter-relationships between multiple factors and variables [27]. It is a method for measuring the relevance between one event and every other one in turn by judging degree of similarity or dissimilarity [28]. Grey relational analysis (GRA) provides a useful tool for problems of limited and superficially rules, and for searching primary relationships among the influential factors and determining important factors that significantly affect the defined objectives [28,29]. This paper develops a methodology called AHP- Grey Correlation Analysis which is a combination of AHP and Grey Correlation Analysis.

#### 2.1.1. Hierarchical Structure of the Project

**Figure 1:**
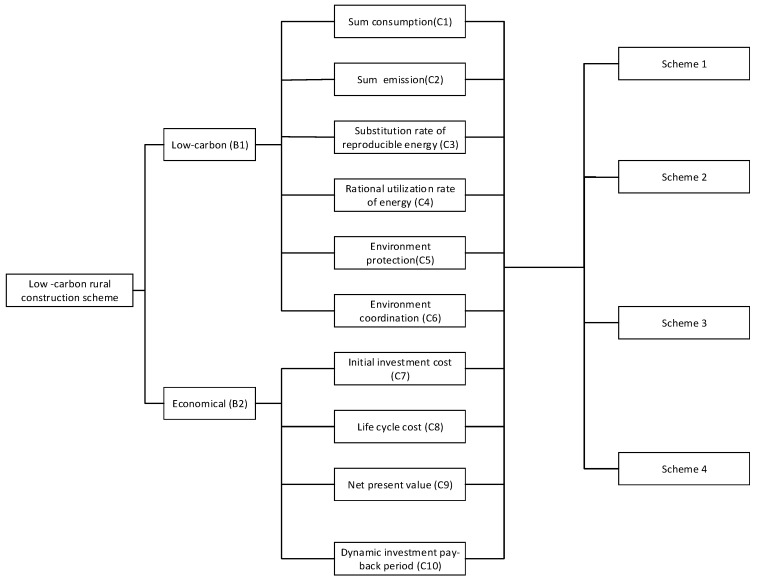
The hierarchy of the AHP model for the selection of an appropriate rural construction structures.

It is essential to set up a hierarchical structure for analyzing the problem in detail. In this paper, we will use three levels, including objective level, criteria level and alternative level. The objective level is the aim to be achieved or the problem to be solved. Two main parts included in the criteria are main criteria and sub-criteria. The alternative level is the level where the choice made is the best. To meet the current requirement of low carbon development and the economic condition of rural residents in China, Low-carbon and Economical were identified out as the main criteria, and for both of them, specific sub-criteria were composed according to the existing low-carbon building research. Here, these sub-criteria brought criteria such as carbon emission, energy consumption, environment protection, etc. into consideration; thus, they were comprehensive and representative [30,31]. The main criteria and sub-criteria for the selection are summarized in Table 1. Therefore, the hierarchy of the AHP model for the selection of an appropriate low-carbon rural construction structure can be obtained and shown as in Figure 1.

#### 2.1.2. Determining the Decision-Making Matrix

There is more than one scheme for us to achieve our final aim, so we suppose that the number of the alternatives can be defined as $D_{i}(i=1,\;2,\;\ldots \;,\;m$). Moreover, these schemes include a lot of indicators defined as $d_{j}\left(j=1,2\ldots ,n\right)$. Then, in the matrix, m is the number of the scheme and n is the number of the targets that influence the decision. After that, $D_{0}$ denotes the best solution for this problem that is not included in $D_{i}$. It is also recognized as a positive ideal result, because all the indicators included are the best. Finally, the decision-making matrix which composed by $D_{i}$ and $D_{0}$ including *m* + 1 schemes can be determined as follows.

(1) $$\bar{D}=\left[\begin{array}{c} D_{1}\\ D_{2}\\ \cdots \\ D_{m}\\ D_{0} \end{array}\right]=\left[\begin{array}{cccc} d_{11} & d_{12} & \cdots & d_{1n}\\ d_{21} & d_{22} & \cdots & d_{2n}\\ \cdots & \cdots & \cdots & \cdots \\ d_{m1} & d_{m2} & \cdots & d_{mn}\\ d_{1}^{0} & d_{2}^{0} & \cdots & d_{n}^{0} \end{array}\right]$$

#### 2.1.3. Normalizing the Decision-Making Matrix 

To decrease the influence caused by different criterion of different schemes, Equation (2) is used for the normalization for the benefit type of the criteria and Equation (3) is used for the cost type of the criteria.

(2) $$S_{ij}=\frac{d_{ij}}{d_{j}^{0}}$$

(3) $$S_{ij}=\frac{d_{j}^{0}}{d_{ij}}$$

#### 2.1.4. Establishing the Weights

The AHP methodology is employed to calculate the weights. First, construct the pairwise comparison matrix considering the decision criteria with the diagonal elements being 1. Next, use the decision makers’ pairwise judgments to fill the comparison matrix with the values in Table 2. Using the comparison judgment, a pairwise comparison matrix is formed, as seen in Equation (4) [29].

(4) [1a12a13a1j⋯a1n1a121a23a2j⋯⋯1a131a231a3j⋯⋯1a1j1a2j1a3j1⋯⋯⋯⋯⋯⋯⋯ajn1a1n⋯⋯1ajn⋯1]

Finally, rank the preference order. A set of alternatives now can be ranked by the root method consists of the following steps:

Step 1: Calculating the product of every line in the matrix with Equation (5)

(5) $$M_{i}=\prod_{j=1}^{n}a_{ij},i=1,2,\cdots ,n$$

where $a_{ij}$ represents the values in the comparison matrix.

Step 2: Calculating the nth root of $M_{i}$.

(6) $$\bar{W_{i}}=\sqrt[{n}]{M_{i}}$$

Step 3: Normalizing the vector $\bar{W}$ ($\bar{W}=\left[\bar{W_{1}},\bar{W_{2}},\bar{W_{3}},\bar{W_{4}},\bar{W_{5}}\right]$) with Equation (6). Then the vector of weight W $\left(W={\left[W_{1}W_{2}\ldots W_{N}\right]}^{T}\right)$ can be obtained:

(7) Wi¯=Wi¯∑j=1nWj¯

#### 2.1.5. Calculating the Relation Coefficient

According to the GRA, the relation coefficient $S_{i}$ of the pair-wise comparison can be calculated by Equation (8)

(8) $$r_{ij}=\frac{{min}_{i}{min}_{j}\left(S_{j}^{0}-S_{ij}\right)+p{max}_{i}{max}_{j}\left(s_{j}^{o}-s_{ij}\right)}{\left(s_{j}^{o}-s_{ij}\right)+p{max}_{i}{max}_{j}\left(s_{j}^{o}-s_{ij}\right)}$$

where p is the distinguishing coefficient [32] (0<p<1).

#### 2.1.6. Calculating the Relational Grade

The overall evaluation of the multiple variables is based on the grey relational grade, which is computed by the grey relational coefficient corresponding to each performance characteristic and the weight coefficient obtained by AHP methodology can be calculated by Equation (9):

(9) $$u_{i}=\sum_{j=1}^{n}w_{j}r_{ij}$$

## 3. Case Study

### 3.1. Basic Information for Four Schemes

To demonstrate the advantage of SIP building, a case study in a rural area is given based on the method proposed in Section 2. The alternatives are selected from the architectural structures used in China. Brick-concrete structures and timber-framed structures are the main ways of constructing houses, and light-steel is the most popular scheme for factories in rural China. SIP building is a new scheme, which is more suitable for sustainable development. So, these four structures are selected to be compared for the case study. Based on the Chinese Engineering Quota, China building code, basic information about the material, and existing research [33,34], the data needed for case comparison can be calculated and gained. Economic criteria such as the initial investment for each structure or scheme are shown in Table 3; Table 4 is obtained from Chinese Engineering Quota and formula of engineering economy. Other basic information which is presented in Table 4 and Table 5, including thickness of outer wall, is collected from material properties such as heat transfer coefficient of outer wall and the China building code.

In the present comparative study, the following four common assumptions were made for consistency. First, the area of the construction is 320 $m^{2},$ with two floors. Second, initial investment only represents the cost of building the structure. In other words, taxes and other additional costs are not included. Third, the same investment of these projects is not covered in the life cycle cost, such as windows, doors, refined decoration and so on. Finally, four buildings, located in both warm-summer and cold-winter areas of China were selected for our case study, with a designed lifespan of 50 years.

### 3.2. Building Up Hierarchical Structure 

**Figure 2:**
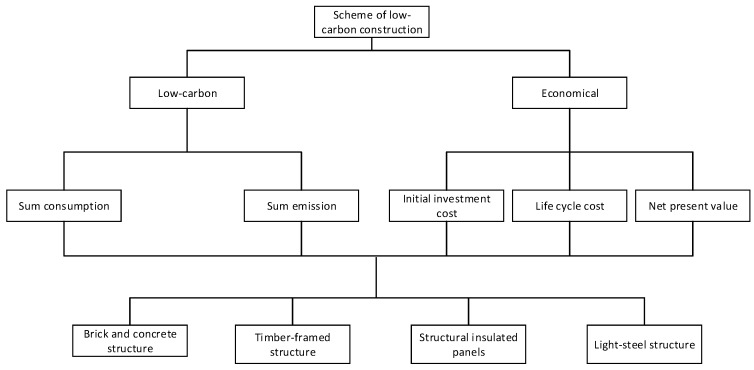
Hierarchical structure of this case.

According to the criteria and sub-criteria listed in Table 1 and the hierarchy described in Figure 1, we set up a new hierarchy model that is suitable for the construction, as shown in Figure 2. Based on the data and statistics collected, the indices needed for the calculations in this section are summarized in Table 6.

From Table 6, we can see that brick-concrete building is the most expensive while timber-framed house is the cheapest in terms of the life cycle cost and the different heat transfer coefficients of outer wall, which is an important factor in power consumption. For the other two schemes, the life cycle cost is similar, which for the SIP building is 33.03 RMB per square meter while light-steel structure is 33.25 every year. However, the comparison results are different in terms of dynamic investment pay-back period and net preset. This proves that the results of SIP building structure are the best and those of the light-steel structure are the worst. It means that, in terms of saving money in the life cycle of the houses, SIP building is the best choice for most rural residents.

### 3.3. The Decision-Making Matrix

It is difficult to ensure the ideal index of each criteria for the schemes because of the complexity and scale of the construction and the differences between the indicators. So, in this paper, the integer of optimal value in each standard is adopted to dispose primary data. Because grey correlation analysis is used to describe the similarity of related factors, the final result can not be influenced by the definition of the ideal norm. Thus, we obtain the decision-making matrix as following.

(10) $$\bar{D}=\left[\begin{array}{c} D_{1}\\ D_{2}\\ D_{3}\\ D_{4}\\ D_{0} \end{array}\right]=\left[\begin{array}{ccccc} 1560 & 3890 & 24 & 41.98 & 0\\ 252 & 628 & 35.2 & 30.32 & 19,978.9\\ 158 & 394 & 33.6 & 35.12 & 38,834.7\\ 208 & 519 & 35.48 & 30.32 & 11,003.6\\ 150 & 390 & 20 & 30 & 40,000 \end{array}\right]$$

### 3.4. Normalizing the Decision-Making Matrix

We normalize the decision-making matrix (10) according to the maximum net present value. Then we can get the result by the Equations (2) and (3)

(11) $$S=\left[\begin{array}{ccccc} 0.096 & 0.100 & 0.833 & 0.715 & 0\\ 0.595 & 0.621 & 0.568 & 0.989 & 0.499\\ 0.949 & 0.990 & 0.595 & 0.854 & 0.971\\ 0.721 & 0.751 & 0.558 & 0.902 & 0.275 \end{array}\right]$$

### 3.5. Calculating the Weight

According to hierarchical structure in Figure 2, the importance assessment of criteria to goal level and alternatives to criteria level are conducted using a suitable scale based on Table 2. Results of the pairwise comparison are obtained by Equation (4). Then, the results are summarized in Table 7, Table 8 and Table 9. Furthermore, the weight of each criterion is provided in these tables, as calculated by Equation (7). After ranking the preference order, the weight can be calculated as Equation (12).

(12) $$W=\left[\begin{array}{ccccc} 0.125 & 0.042 & 0.216 & 0.208 & 0.364 \end{array}\right]$$

**Figure 3:**
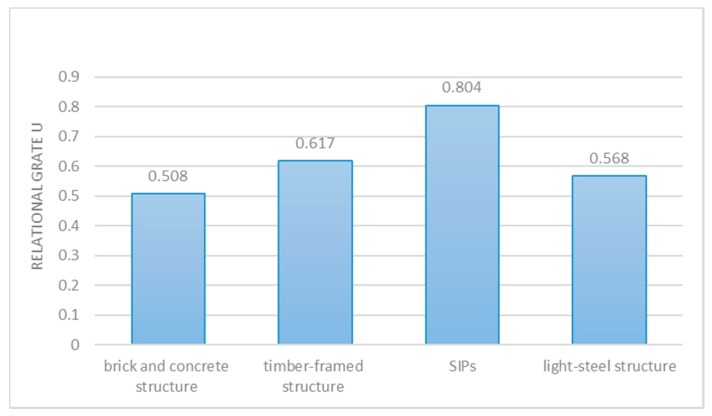
Relational grade U of each scheme.

Then the grey relational grade is calculated according to Equation (8). The results of the grey relational coefficient are showed in the matrix R. Finally, the relational grades computed by Equation (9) are listed in Table 10 and shown in Figure 3.

(13) $$S=\left[\begin{array}{ccccc} 0.354 & 0.356 & 0.749 & 0.636 & 0.332\\ 0.551 & 0.568 & 0.535 & 0.980 & 0.498\\ 0.908 & 0.982 & 0.551 & 0.774 & 0.947\\ 0.641 & 0.667 & 0.529 & 0.836 & 0.407 \end{array}\right]$$

Table 10 and Figure 3 described the results of both economy and total carbon emission calculated by AHP-grey correlation analysis, which confirms that SIP buildings are the most sustainable building structure in rural areas, of the four altefigurrnatives.

## 4. Discussion

This study compared the performance of four different building structures (brick and concrete structures, SIPs, timber-framed and light-steel structures). Our findings are further discussed here.

Before conducting a comprehensive analysis of economy and carbon emission in SIP building, an analysis could first be done from these two perspectives. First of all, we only consider the economic factors. According to the basic information and the results of economic evaluation in Table 3 and Table 6, we can see that, though the investment of brick-concrete building is the lowest, its life cycle cost is much higher than the other three alternatives. This is because brick and concrete structures lead to the highest energy and material consumptions. According to the results of economic evaluation, SIP building is better than the other schemes in most indices, so it is most economical for rural residents to choose SIP building.

For carbon emission, as shown in Table 6, building with SIPs has lower embodied emissions due to low carbon emission during materials production, which is the largest contribution to the embodied emissions, as shown in previous studies [35,36,37]. Furthermore, the transfer coefficient of material can influence the consumption of fossil fuels, so greenhouse gases of the other three schemes caused by fossil fuels are larger than in SIPs. In short, SIP building is the best building structure to reduce carbon emission during construction and energy consumption.

According to the results of a comprehensive analysis for economy and carbon emission in SIP buildings, shown as Table 10, the investments and the life cycle cost of SIP building is not the lowest. However, as previous research shows, SIPs provide several beneficial features, such as excellent thermal and acoustic performance, environmental friendliness, ease of erection, and off-site construction [36]. Therefore, the construction period of SIP building can be dramatically reduced, which in turn decreases carbon emission and investment cost.

Recently, China’s 13th Five-year Plan for the Construction Industry emphasized that “energy conservation and emission reduction could become the new growth point of the construction industry”, which reflects that the Chinese construction industry needs to be transformed towards sustainability and supporting China's future development from the aspects of low-carbon emission, economic development and environment protection. Based on this policy, and according to the results of the case study, the conclusion that SIP building is better than the other three schemes for sustainable construction in China can be obtained, and this conclusion is also consistent with the results of existing study [38].

## 5. Conclusions

This paper presents a methodology (AHP-Grey Correlation Analysis) to identify appropriate building structures in rural China using the criteria of economic benefit and low carbon emission. AHP is used to determine the weights of the decision criteria and Grey Correlation Analysis is used to rank the alternatives. Four possible rural house structures have been studied in our illustrative case study. The results in this paper clearly demonstrate the advantages of SIPs compared to the other alternatives, including brick and concrete structure, timber-framed structure, and light-steel structure.

As rural residents may lack understanding about SIPs, extensive promotion and education could be implemented in rural areas. It is strongly advised that governments popularize SIPs through the media, magazine, billboards, and demonstrative projects. However, the initial investment of SIPs is higher than that of a brick and concrete structure, which is currently the most popular building structure in rural China. Thus, the central and local governments can offer more financial incentives to support those residents who desire the SIP building structure.

This research mainly focuses on the criteria of low carbon emission and economic benefit in rural China, and proves the advantages of the SIP building structure. Adopting this structure in rural China can have a great potential of reduction in carbon emission.

## Figures and Tables

**Table 1:** Criteria and sub-criteria used in the AHP model.

Criteria	Sub-Criteria
Low-carbon ($B_{1}$)	Sum consumption ($C_{1}$)
	Sum emission ($C_{2}$)
	Substitution rate of reproducible energy ($C_{3}$)
	Rational utilization rate of energy ($C_{4}$)
	Environment protection ($C_{5}$)
	Environment coordination ($C_{6}$)
Economical ($B_{2}$)	Initial investment cost ($C_{7}$)
	Life cycle cost ($C_{8}$)
	Net present value ($C_{9}$)
	Dynamic investment pay-back period ($C_{10}$)

**Table 2:** Verbal judgment of preference and numerical rate.

Verbal Judgment of Preference	Numerical Rate
Equal importance	1
Moderately more importance than another	3
Essentially or strongly more importance than another	5
Demonstrably more importance than another	7
Absolutely more importance than another	9
Intermediate values between the two adjacent judgments	2, 4, 6, 8

**Table 3:** Initial investment of each schemes (unit: ¥).

Scheme 1: Brick and Concrete Structure	Scheme 2: Timber-Framed Structure	Scheme 3: SIPs	Scheme 4: Light-Steel Structure
750	1100	1050	1120

**Table 4:** Cost for supporting the house.

Structure	Power Consumption (Kilowatt/m^2^)	Electric Rates (¥)	Other Rates (¥)	Annual Operating Cost (¥)
Brick and concrete structure	14.96	2776.58	7211.72	9987.07
Timber-framed structure	2.95	547.52	1066.24	1613.76
SIPs	2.29	425.02	1007.56	1432.58
Light-steel structure	3.08	571.65	1206.4	1778.05

**Table 5:** The other information of the material.

Structure	Heat Transfer Coefficient of Outer Wall [W/(m^2^·K)]	Refrigeration and Heating Time (h)	Thickness of Outer Wall (mm)
Brick and concrete structure	K1 = 1.66	8	240
Timber-framed structure	K2 = 0.33	8	130
SIPs	K3 = 0.256	8	160
Light-steel structure	K4 = 0.345	8	160

**Table 6:** The data of each index.

	Index	Total Energy Lost	Total Emission (t)	Initial Investment (10 k ¥)	Life Cycle Cost (¥/m^2^·Year)	Dynamic Investment Pay-Back Period (Year)	Net Present Value (¥)
Structure							
Brick and concrete structure		1560	3890	24	41.98	-	-
Timber-framed structure		252	628	35.2	30.32	27.85	19,978.9
Structural insulated panels (SIPs)		158	394	33.6	33.03	19.21	38,834.7
Light-steel structure		208	519	35.84	33.25	34.41	11,003.6

**Table 7:** Goal-level criteria level A-B and the weight W.

A	B_1_	B_2_	W
B_1_	1	1/5	0.167
B_2_	5	1	0.833

**Table 8:** Criteria-level sub-criteria level B_1_-C and the weight W.

B_1_	C_1_	C_2_	W
C_1_	1	3	0.75
C_2_	1/3	1	0.25

**Table 9:** Criteria-level sub-criteria level B_2_-C and the weight W.

B_2_	C_3_	C_4_	C_5_	W
C_3_	1	5/4	5/7	0.313
C_4_	4/5	1	4/7	0.250
C5	7/5	7/4	1	0.437

**Table 10:** The relational grade of each scheme.

Scheme	Brick and Concrete Structure	Timber-Framed Structure	SIPs	Light-Steel Structure
Relational grade U	0.508	0.617	0.804	0.568
